# Whole genome characterization of autochthonous *Bos taurus brachyceros* and introduced *Bos indicus indicus* cattle breeds in Cameroon regarding their adaptive phenotypic traits and pathogen resistance

**DOI:** 10.1186/s12863-020-00869-9

**Published:** 2020-06-22

**Authors:** Archile Paguem, Babette Abanda, Mbunkah Daniel Achukwi, Praveen Baskaran, Stefan Czemmel, Alfons Renz, Albert Eisenbarth

**Affiliations:** 1 Department of Biological Science, Faculty of Science, University of Ngaoundéré, Ngaoundéré, Cameroon; 2 Department of Comparative Zoology, Institute for Evolution and Ecology, University of Tübingen, Tübingen, Germany; 3 TOZARD Research Laboratory, P.O. Box 59, Bambili-Tubah, Bamenda, Cameroon; 4 Quantitative Biology Center (QBiC), University of Tübingen, Tübingen, Germany; 5 Institute of Novel and Emerging Infectious Diseases, Friedrich-Loeffler-Institut, Federal Research Institute for Animal Health, Insel Riems, Greifswald, Germany

**Keywords:** Whole genome sequencing, Trypanotolerance, *Bos taurus:* Namchi, Kapsiki, *Bos indicus*: white Fulani, Red Fulani, Gudali, Cameroon

## Abstract

**Background:**

African indigenous taurine cattle display unique adaptive traits shaped by husbandry management, regional climate and exposure to endemic pathogens. They are less productive with respect to milk and meat production which has been associated with amongst others, small size, traditional beliefs, husbandry practices, limited feed resources, disease burden and lack of sustained breeding for trait improvement. This resulted in the severe dwindling of their population size rendering them vulnerable to extinction.

The Namchi taurine cattle breed is referred to as [Namchi (Doayo)] and shows resistance traits against trypanosome infection and exposure to tick infestation. Nonetheless, the historically later introduced Zebu cattle are the main cattle breeds in Africa today, even though they suffer more from locally prevailing pathogens.

By using a whole genome sequencing approach, we sequenced with high depth for the first time the genomes of five cattle breeds from Cameroon in order to provide a valuable genetic resource for future African cattle breeding: the Namchi, an endangered trypano-tolerant taurine breed, the Kapsiki, an indigenous trypano-susceptible taurine breed, and three Zebu (*Bos indicus indicus*) breeds: Ngaoundere Gudali, White Fulani and Red Fulani.

**Results:**

Approximately 167 Gigabases of raw sequencing data were generated for each breed and mapped to the cattle reference genomes ARS-UCD1.2 and UMD3.1.The coverage was 103 to 140-fold when aligning the reads to ARS-UCD1.2 with an average mapping rate of ~ 99%, and 22 to 30-fold when aligning the reads to UMD3.1 with an average mapping rate of ~ 64%. The single nucleotide polymorphisms (SNPs) obtained from analysis using the genome ARS-UCD1.2 were compared with reference genomes of European *Bos taurus* Holstein, the Asian *Bos indicus* Brahman, and the African trypanotolerant N’Dama breeds.

A total of ~ 100 million (M) SNPs were identified and 7.7 M of those were breed-specific. An approximately 11.1 M constituted of small insertions and deletions. By using only breed-specific non-synonymous variants we identified genes as genetic signatures and associated Gene Ontology (GO) terms that could explain certain cattle-breed specific phenotypes such as increased tolerance against trypanosome parasites in the Namchi breed and heat tolerance in the Kapsiki breed. Phylogenetic analysis grouped, except for Namchi, the *Bos taurus* breeds Kapsiki, N’Dama and Holstein together while the *B. indicus* breeds White and Red Fulani, Gudali and Brahman clustered separately. The deviating result for Namchi indicates a hybrid status of the selected animal with a recent introgression of Zebu genes into its genome.

**Conclusions:**

The findings provide the first comprehensive set of genome-wide variant data of the most important Cameroonian cattle breeds. The genomic data shall constitute a foundation for breed amelioration whilst exploiting the heritable traits and support conservation efforts for the endangered local cattle breeds.

## Background

More than 150 cattle breeds or distinct populations have been recorded in Africa [1, 2]. Their phenotypes cluster into the humpless taurine, the humped Zebu, and the anciently fixed taurine-Zebu crossbreeds known as Sanga in East Africa [3].

In Sub-Saharan Africa, trypanosomiasis (Nagana), dermatophilosis, tick-borne diseases and gastrointestinal helminthiasis are the major endemic diseases affecting cattle productivity [4, 5]. Indigenous local taurine breeds like Doayo (also known under the Fulani word Namchi) are more resistant or tolerant to most endemic diseases than Zebu cattle [5]. They originated from ancestral aurochs populations *Bos primigenius primigenius* and *B. primigenius opisthonomus* from two centers of domestication, namely the Middle East and North Africa, respectively [6, 7].

Today Namchi and Kapsiki are geographically restricted to endemic areas of human and animal trypanosomiasis in Northern Cameroon. Whereas N’dama and Kuri cattle are grouped as residual longhorn *Bos taurus longifrons* introduced already 10,000 years ago [5, 8], Baoulé, Namchi and Kapsiki belong to the West African Shorthorn (WAS) *Bos taurus brachyceros* domesticated on the continent some 6500 years ago [6, 7].

The Kapsiki cattle form a population of approximately 5000 animals that are found mainly in the Mayo Tsanaga (Rhumsiki) area of the Far North region [9]. In contrast, the Namchi cattle have a population size of only 1000 to 2000 heads in the Poli mountains, which are up to 1900 m above sea level- and surround savannah lowlands in the Faro division of Cameroon’s North region [8, 10]. The breed is well adapted to the local environment, including endemic parasites like trypanosomes and ticks [8, 11], but of small size and weight, thus economically not interesting for milk and meat production. The usually small herd size of 5 to 50 animals are kept semi-wild, and are neither milked nor exploited commercially. They rather play an important role in the traditional culture of local tribes, like dowries, special feasts and rituals. During the last three decades, uncontrolled crossbreeding with Zebu cattle have severely dwindled the gene pool of this taurine cattle population [8]. In 1992, these breeds have been classified by the Food and Agricultural Organization (FAO) as being at risk of becoming extinct [10], hence the conservation of their genetic resources has been highly prioritized. Unfortunately, the majority of the planned strategies for their conservation has not been adopted in the field. The continuous influx of Zebu genes into the WAS breeds threatens the innate characteristics of trypanotolerance and other disease resistances [3].

*Bos indicus* Zebu cattle in Africa fall into two distinct groups, the West African Zebu (WAZ) and East African Zebu (EAZ). In Cameroon, 99% of the estimated population of six million cattle are WAZ breeds. They consist of two major sub-types of the Sokoto and Adamawa Gudali [12]. In Central Africa, they have the highest potential for beef and dairy production in comparison to other regional WAZ breeds, like White Fulani and Red Fulani. These Fulani cattle are long-horned and long-legged Zebu cattle and are mainly kept by the nomadic Bororo people [13]. All Zebu breeds were introduced through the Nile-valley and the Horn of Africa around 2000 years ago. They started to become more widespread about 700 years ago with hamitic migrations in North and East Africa [7, 14] and throughout the Sahel zone south of the Sahara. They arrived in Northern Cameroon, coming from the Bornu (Nigeria today) some 200 years ago. This relatively short time span for evolutionary adaption is reflected by a higher susceptibility to locally endemic diseases and disease vectors making reliance on veterinary drug interventions essential for their survival.

Better knowledge of unique adaptive traits against locally prevailing pathogens is needed not only for breed conservation, but also for future genetic amelioration of cattle breeds to mitigate food insecurity problems in Africa. Long-term selection pressure has operated on the genomic architecture and on regions that control traits for adaptive fitness [1]. For example, autosomal and Y-chromosomal microsatellites indicate a high level of genetic diversity in African cattle breeds as a consequence of repetitive introgression of Zebu genes into autochthonous taurine genome across the continent [1–8]. Genome research initiatives, like Bovine Genome Sequencing, HapMap and 1000 Bulls have fostered our understanding of bovine evolution and the complex formation of genetic variants [15–17]. The free availability of cattle reference genomes facilitates whole genome re-sequencing approaches, which are steadily expanding [15–17].

In this study, we characterize for the first time the complete genomes of five cattle breeds in Cameroon, namely the endangered taurine trypanotolerant Namchi, the trypano-susceptible Kapsiki taurine, and the three Zebu breeds Gudali, White Fulani and Red Fulani, which are all trypano-susceptible. Using the genomic data, ~ 100 million (M) SNPs were identified in this study of which 7.7 M (~ 8%) were considered as novel variants. In general, lower genetic diversity was found in African taurine cattle breeds than in the Cameroonian *Bos indicus* breeds. Furthermore, breed-specific non-synonymous variants were detected, which can be linked to important traits such as trypanotolerance in Namchi and heat tolerance in Kapsiki.

## Results and discussion

### Whole genome sequencing analysis

Genomic DNA from the cattle breeds Gudali, White Fulani, Red Fulani, Namchi and Kapsiki were sequenced (150-bp paired-end reads) with the Illumina HiSeq4000 sequencing platform and libraries were sequenced using 150-bp paired-end reads. This generated a total of ~ 840 Gb of raw reads for all five breed samples analyzed together, averaging to ~ 167 Gb per sample which provides, to the best of our knowledge, the first comprehensive set of high depth, whole genome variant data of these breeds.

**Fig. 1:**
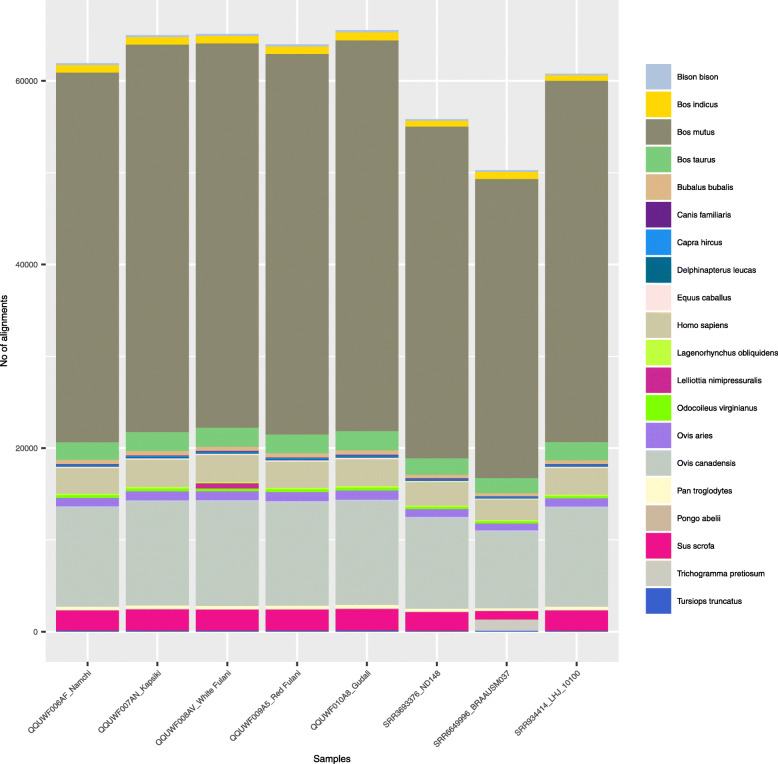
Pairwise alignment of contigs assembled from unmapped reads to the non-redundant nucleotide database from NCBI. Each bar represents an individual cattle breed and contained the twenty most common species with significant alignments to the de novo assembled contigs

The chosen approach of high depth sequencing yielded approximately 10^9^ reads per sample (Tables 1 & 2) which allowed us to obtain a high coverage per animal tested. However, it also resulted in a relatively low mapping rate for the African cattle breeds ranging from 63 to 65% when aligned to the reference genome UMD3.1 (Table 1). This low mapping rate could be explained by 1) the PCR-free preparation of sequencing libraries, which implies that bovine DNA and non-bovine DNA such as blood microbes and parasites could have been sequenced at similar rates, or 2) the reference genome is incomplete, or 3) the African cattle breed samples chosen are evolutionarily more distant compared to the reference genome and therefore contain sequences of genomic regions not present in the UMD3.1 cattle reference genome. In order to better understand this, unmapped reads were assembled into contigs using the de novo sequence assembler ABySS and compared against the NCBI Blastn database [Fig. 1, Additional file 1 Table S1]. The results obtained from this analysis did not support the hypothesis of microbial or parasitic DNA contamination. Species such as *Trichogramma pretiosum* in the Brahman control sample, the bacteria *Lelliottianimi pressuralis* and *Enterobacter* spp. in White Fulani, *Babesia* spp. and *Theileria spp.* cosmopolitan blood parasites of ruminants which are known to inflict diseases were detected in Namchi, but only supported by a very low number of Blastn alignments [Additional file 1 Table S1]. Still, the presence of such organisms in our samples is in line with a recent epizootiological survey in the same indigenous Cameroonian cattle breeds that revealed nearly 90% of animals carried tick-borne bacterial, piroplasmid and protozoan pathogens [18, 19].

**Table 1:** Summary of sequencing results of the genomes of five Cameroonian cattle breeds including the number of total reads and variants called in million (M) reads

Breeds	Mapped Reads	Total Reads	Mapping rate (%)	Coverage [x]	SNPs	Indels	Bs- SNPs	Hom	Het	Het/ Hom
Namchi	596.3	935.3	63.7	22.8	6.31	0.53	0.40	2.51	3.80	1.5
Kapsiki	743.7	1160.6	64.1	28.6	5.40	0.47	0.37	1.55	3.85	2.5
W. Fulani	707.6	1103.1	64.1	27.2	6.42	0.55	0.42	2.29	4.13	1.8
R. Fulani	716.3	1102.2	65.0	27.6	6.70	0.57	0.47	2.15	4.55	2.1
Gudali	804.9	1271.1	63.3	30.8	6.65	0.57	0.46	2.17	4.49	2.1
N’Dama	154.5	282.1	54.8	4.7	4.26	0.35	0.22	1.53	2.73	1.8
Brahman	146.4	177.0	82.7	5.1	7.31	0.60	0.76	2.96	4.36	1.5
Holstein	255.7	460.6	55.5	7.6	3.05	0.26	0.33	1.19	1.87	1.6

The reference genome breed was Hereford (UMD3.1). Whole genome data of the breeds N’Dama, Brahman and Holstein were retrieved from the NCBI archive SRA [Holstein (SRR934414), N’Dama (SRR3693376) and Brahman (SRR6649996)]. Hom = homozygous, Het = heterozygous, Het/ Hom = heterozygous to homozygousratio, W. Fulani = White Fulani; R. Fulani = Red Fulani. Bs-SNPs = breeds specific SNPs

**Table 2:** Summary of sequencing results of the genomes of five Cameroonian cattle breeds including the number of total reads and variants called in million (M) reads

Breeds	Mapped Reads	Total Reads	Mapping rate (%)	Coverage [x]	SNPs	Indels	Bs-SNPs	Hom	Het	Het/ Hom
Namchi	930.8	935.3	98.9	102.8	12.74	1.51	0.91	5.54	8.10	1.5
Kapsiki	1154.9	1160.6	99.0	127.6	10.81	1.34	0.83	3.42	8.23	2.4
W. Fulani	1098.0	1103.1	99.0	121.3	12.99	1.56	0.95	5.07	8.87	1.7
R. Fulani	1098.0	1102.2	99.1	121.3	13.54	1.62	1.05	4.73	9.86	2.1
Gudali	1267.0	1271.1	99.1	140.0	13.37	1.62	1.09	4.77	9.70	2.0
N’Dama	271.8	282.1	99.6	30.0	8.53	1.01	0.48	3.32	5.68	1.7
Brahman	176.9	177.0	99.4	19.5	14.21	1.73	1.70	6.53	9.38	1.4
Holstein	453.1	460.6	99.5	50.0	5.66	0.76	0.73	2.59	3.80	1.5

The new reference genome breed was Hereford (ARS-UCD1.2). Whole genome data of the breeds N’Dama, Brahman and Holstein were retrieved from the NCBI archive SRA [Holstein (SRR934414), N’Dama (SRR3693376) and Brahman (SRR6649996)]. Hom = homozygous, Het = heterozygous, Het/ Hom = heterozygous to homozygous ratio, W. Fulani = White Fulani; R. Fulani = Red Fulani. Bs-SNPs = breeds specific SNPs

Rather, the mapping results indicated that the analyzed breeds are evolutionary more distant compared to the reference genome UMD3.1, or that this genome is not complete. This assumption is supported that *Bos mutus* was the best scoring result in 65% of the Blastn alignments with a mean sequence identity of 98% across all samples, indicating that most unmapped read contigs were of Bovidae origin, but have not been found in the reference genome UMD3.1. In contrast, *Bos taurus* and *Bos indicus* reads were only found in ~ 3% and ~ 1% of the Blastn hits, respectively, demonstrating that most of the reads originating from those species were correctly mapped. There were no obvious differences in Blastn results when comparing African Zebu cattle with Namchi and Kapsiki [Fig. 1, Additional file 1 Table S1], although it seems conceivable to expect Namchi and Kapsiki taurine breeds rather distinct from the reference genome in comparison to the Zebu cattle. The recently published reference genome assembly ARS-UCD 1.2 (NCBI RefSeq accession GCF_002263795.1), based on the same original animal (Hereford breed UMD3.1) was created by applying a combination of long and short reads for a de novo assembly strategy, and showed a > 200-fold improvement in continuity, as well as 10-fold improvement in accuracy and completeness than the previous cattle reference genome [20]. Therefore, this optimized genome was also used as reference to map the reads of the Cameroonian cattle breeds. Interestingly, a very high proportion of raw reads was mapped ranging from 98.9% for Namchi up to 99.6% for Red Fulani (Table 2). Our mapping rates were even higher than reported by Kim et al. [17] from other indigenous East African cattle breeds (Ankole, Boran and Ogaden) and of other cattle re-sequencing studies published [16, 17, 21–23]. Further, the depth of coverage, ranging from 103-fold for *Bos taurus* Namchi to 140-fold for *Bos indicus* Zebu Gudali is also considerably high in comparison to 10.8- and 15.8-fold coverage obtained by Kim et al. [17] and Kawahara-Miki et al. [21], respectively. Taylor et al. [22] suggested that about 95% of the total variants within the genome of cattle are discovered at an average sequence depth of 23.3-fold which implies the data obtained in this study is sufficient to detect SNPs and InDels variants with high confidence.

### Variant calling results

**Fig. 2:**
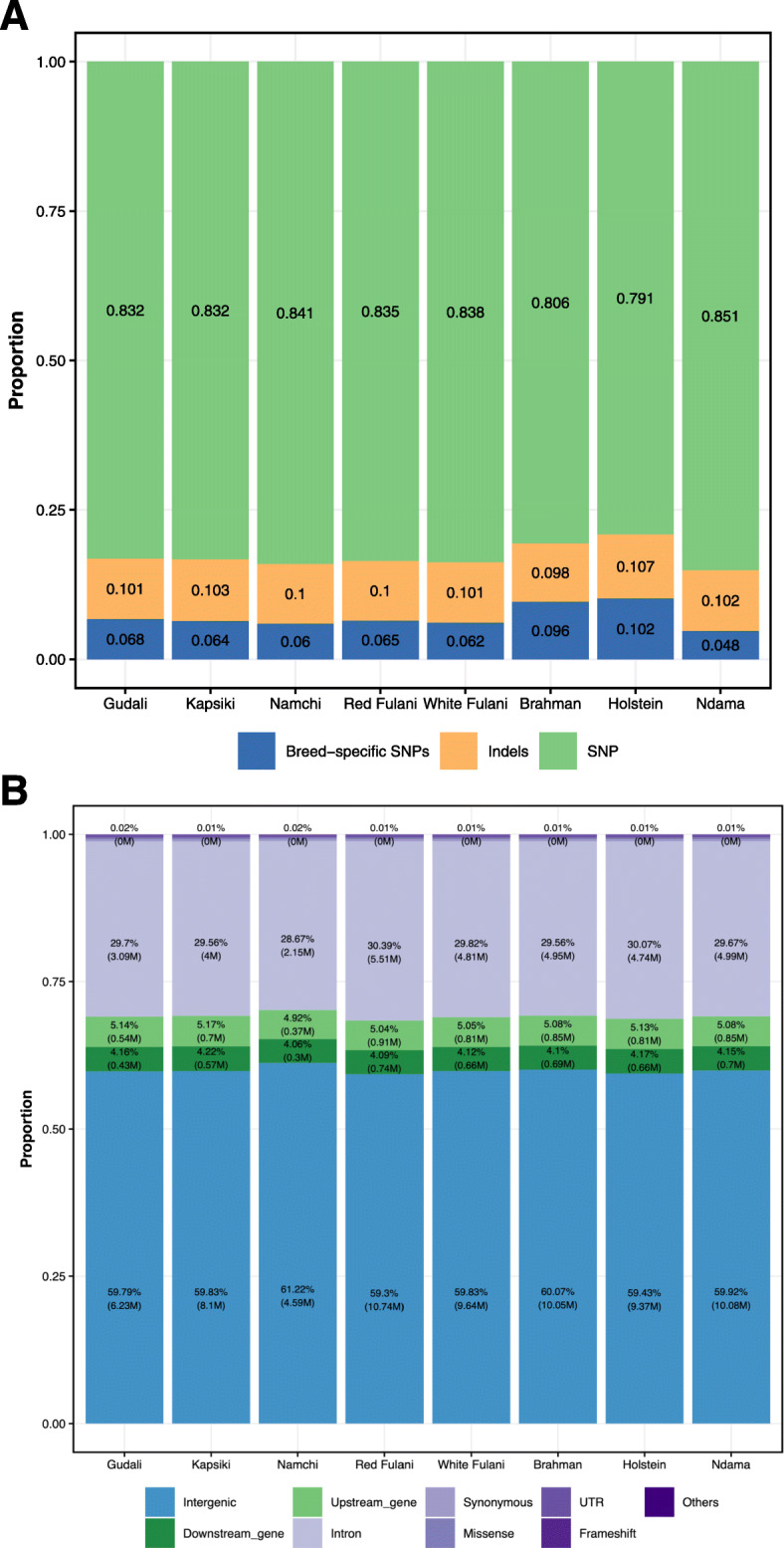
Distribution of variants per breed and genomic features. **A**) Bar plot showing the proportion of common SNPs found in at least two breeds (green), breed-specific SNPs (blue) and InDels (orange) across all the examined breeds. **B)** Bar plot showing numbers in million and proportion of variants types and functional consequences

A total of ~ 100 million (M) SNPs were identified in this study of which 7.7 M (~ 8%) were not found in the 1000 Bulls Genomes Project and considered as novel variants (Table 2; Fig. 2A). On average for each breed, 1.4 M (12%) of the detected variants had small insertions and deletions (InDels, Table 2). The SNP variants results from Cameroonian cattle were much higher as compared to the 27 M SNPs obtained by Stafuzza et al. [23] on *Bos indicus* Gyr, Girolando, Gruzerat and *Bos taurus* Holstein cattle breeds from Brazil, whereas our obtained SNP variants were markedly lower as compared to those reported by Kim et al. [17] on East African zebu (Boran, Ogaden, Kenana) and Sanga (Ankole, taurine/zebu crossbreeds). The ratio of the number of heterozygous to homozygous SNP variants was different across the cattle breeds. Brahman, Holstein and Namchi had the lowest rate, whereas Kapsiki had the highest (Table 2). The low ratio of heterozygous to homozygous SNPs in Brahman and Namchi cattle could mean that they experience admixture, as reported by Freemann et al. [24] in African taurines from Cameroon.

### Genetic variability and similarity across breeds

**Fig. 3:**
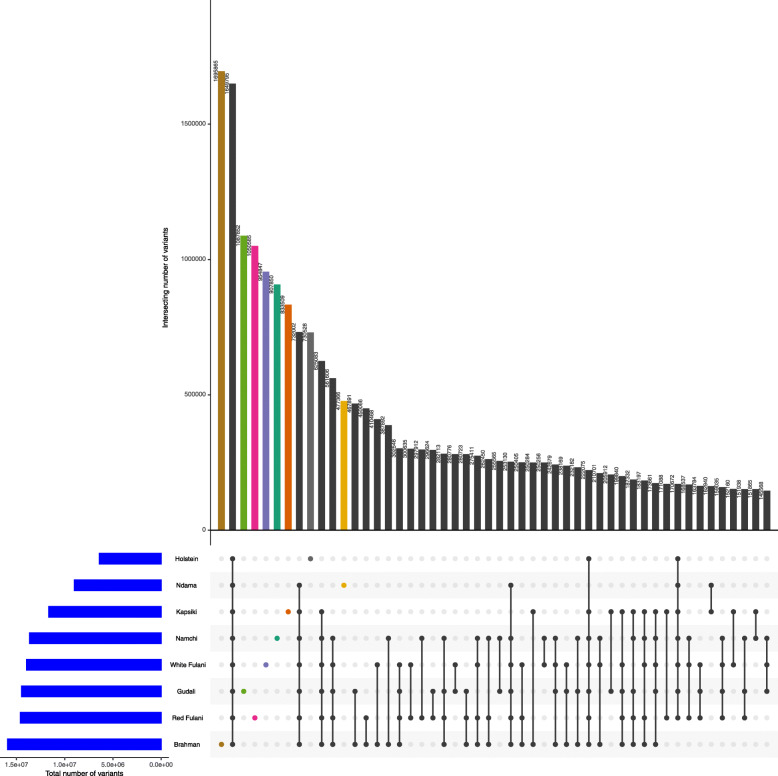
Relationship between the different cattle breeds showing the number of SNPs that are common across different breeds along with the total number of variants (blue) and the number of breed-specific SNPs are as follow: Brahman (pink), Red Fulani (brown), Gudali (green), White Fulani (grey), Namchi (blue), Kapsiki (purple), N’dama (red) and Holstein (orange). The first bar (black) shows the number of SNPs that are found in all eight breed samples

For downstream analysis of single nucleotide polymorphisms (SNPs) we used the genome ARS-UCD1.2 but not UMD3.1 and compared it with reference genomes of European *Bos taurus* Holstein, Asian *Bos indicus* Brahman and African trypanotolerant N’Dama breeds. A total of 1,649,795 SNPs were common across all breeds, and 302,546 SNPs were Zebu-specific, distributed between Brahman, Red Fulani, White Fulani and Gudali cattle breeds (Fig. 3). More surprisingly, there were 27,443 SNPs exclusively shared between the European taurine Holstein and WAS taurine N’Dama, Kapsiki and Namchi, apart from 162,940 SNPs which were shared between N’Dama and Kapsiki only. 151,865 SNPs and 163,784 SNPs were shared between Cameroonian taurine (Kapsiki and Namchi) and Zebu (Red Fulani, Gudali and White Fulani), respectively. Furthermore, 170,672 SNPs were common between all tested cattle breeds except Brahman cattle.

In general, we observed a lower genetic diversity in African taurine cattle breeds than in the Cameroonian *Bos indicus* breeds (Table 2). The highest proportion of breed-specific (bs) SNPs were found in *Bos indicus*: Brahman, Red Fulani, Gudali and White Fulani, respectively, and the lowest breed-specific SNPs were found on taurine breeds N’dama, Holstein, Kapsiki and Namchi, respectively (bs-SNPs are color labelled in Fig. 3). This apparently lower genetic diversity in African taurine breeds was already earlier argued by Kim et al. [17] who linked it to the low effective population size and/or population bottlenecks following fatal disease outbreaks such as Rinderpest. In contrast, indicine Zebu cattle and composites with larger effective population size exhibit a higher level of nucleotide diversity. Furthermore, the higher nucleotide diversity of taurine Namchi and Kapsiki as compared to N’Dama and Holstein may be due to the long history of *Bos indicus* introgression [24, 25].

The density of variants per chromosome was proportional to the chromosome length, except for the X chromosomes which showed a lower number of variants identified (Additional file 2 Fig. S2). These findings were expected because the DNA of X chromosomes is subject to an increased natural selection, which leads to less genetic diversity [23].

### Breed clustering and relationships

**Fig. 4:**
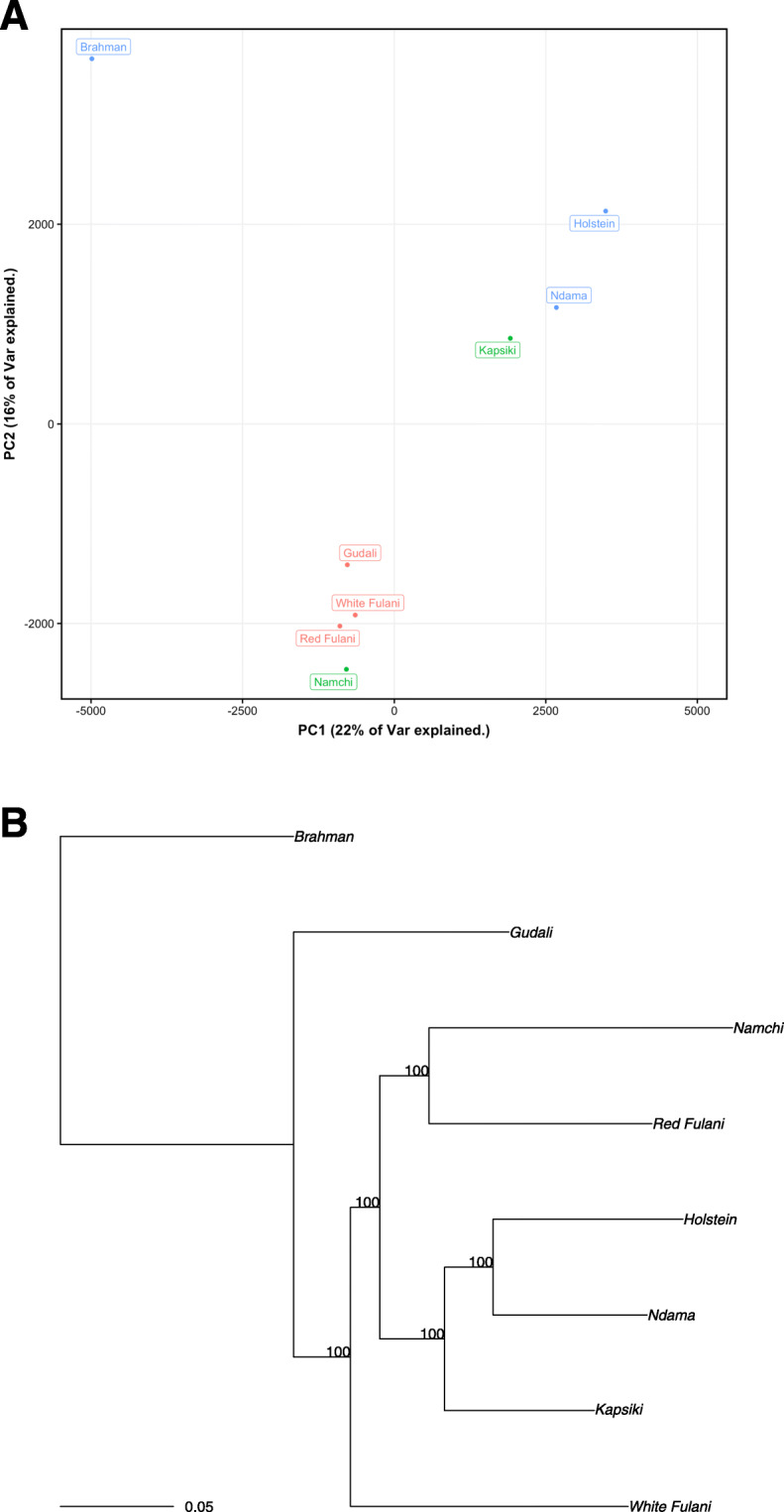
Genomic relationship among cattle breeds. **A)** Principal component analysis using autosomal SNP data only, which shows the distribution of different cattle breeds across the first two principal components. Colors separate the samples into the groups Zebu breeds (red), taurine breeds (green) and the three controls (blue) used in this study. **B)** Phylogenetic maximum likelihood tree of autosomal SNPs variants

The cluster relationship between breeds was analyzed by a principal component analysis (PCA) using all autosomal SNPs (Fig. 4A). The first two principal components explain 22 and 16% of the total variance, respectively. Except for Namchi, the other WAS breeds N’Dama, and Kapsiki form a separate cluster from WAZ breeds. The WAS breeds N’dama, and Kapsiki are also closer to European taurine Holstein than WAZ breeds, and both WAS and WAZ are clearly separated from Zebu Brahman. This indicates the possibility of admixture events between the West African cattle breeds. To further understand the genetic network among those breeds, a phylogenetic tree analysis (Fig. 4B) was carried out with the same autosomal SNPs data as for PCA analysis by using Randomized Accelerated Maximum Likelihood models (RAxML). Again, except for Namchi, the *Bos taurus* breeds Kapsiki, N’Dama and Holstein cluster together while the *B. indicus* breeds White Fulani, Gudali, Brahman clustered on a separate clade. The WAS Kapsiki and Namchi cattle are closer to WAZ cattle as compared to European taurine Holstein. In addition, the WAZ are evolutionary distant to Indian Zebu Brahman. This observation concords with previous studies of WAS indicating they possess admixture with indicine ancestry between 22.7 and 74.1% in Central Africa [26, 27]. Gudali are more closely related to Indian Brahman cattle than White Fulani and Red Fulani (Fig. 4B). The Indian Zebu genes introgression into African Zebu breeds has been reported based on autosomal microsatellite markers between 55 and 83% [3, 27]. The PCA and RAxML findings presented here illustrate the evolution of Cameroonian cattle breeds is distant both to Indian Zebu Brahman and European taurine Holstein. The higher number of heterozygous to homozygous variants ratio in Kapsiki (2.5) than in Namchi (1.5) (Tables 1 & 2) was unexpected, because Kapsiki has been regarded as an indigenous taurine population with highest Zebu gene introgression over the last three decades based on microsatellite data [11, 24, 25]. Namchi and Kapsiki have been classified by Freeman et al. [24] as hybrids rather than pure breeds. The phylogenetic position of Namchi more closely related to Red Fulani than WAS indicated recent Zebu introgression into the genome of Namchi. Although the selected Namchi was not different in appearance to other animals in the region, we cannot exclude whether it has been a product of a recent cross-hybridization with another cattle breed, and thus not representing the pure breed genome. It is reported that there are still some isolated herds of purebred Namchi cattle in the Poli area, but the present study did not have the tools to screen hybridization levels in the selected animal for whole genome data generation. Such screening would be necessary in the present context where traditional farming systems face numerous challenges towards maintaining purely taurine breeds due to rampant cross breeding.

### Functional annotation and gene ontology analysis of high and moderate impact breed-specific SNPs and InDels

The SNPs and InDels were annotated in order to identify the location of the variant in terms of genomic features using the tool snpEFF [28]. In general, all the eight breeds exhibited similar distributions of SNPs and InDels in various genomic annotation categories. Most annotated variants were located in intergenic regions (60%) and introns (30%). The remaining SNPs were found on downstream genes (4%), upstream genes (5%), untranslated regions (UTR) (0.5%), missense (0.6%), frameshift (0.02%) and other areas (0.7%) (Fig. 2B).

Breed-specific variants with high and moderate impact such as frameshift, missense, splice acceptor, splice donor, start lost and stop gained, that may putatively change amino-acids codons are located in and/or close to genes that may lead to functional changes were examined in each chromosome. Overall, 4349 genes with such mutations were identified: Most of them were from the breeds White Fulani and Kapsiki (both > 2000 genes) and the remaining genes were separated on the other breeds as follows: in Red Fulani 11 genes, in Gudali 8 genes, in Namchi 6 genes, and in the three control samples Brahman 27 genes, Holstein 11 genes, and in N’Dama 10 genes, respectively (Additional file 3 Table S3-S10).

**Fig. 5:**
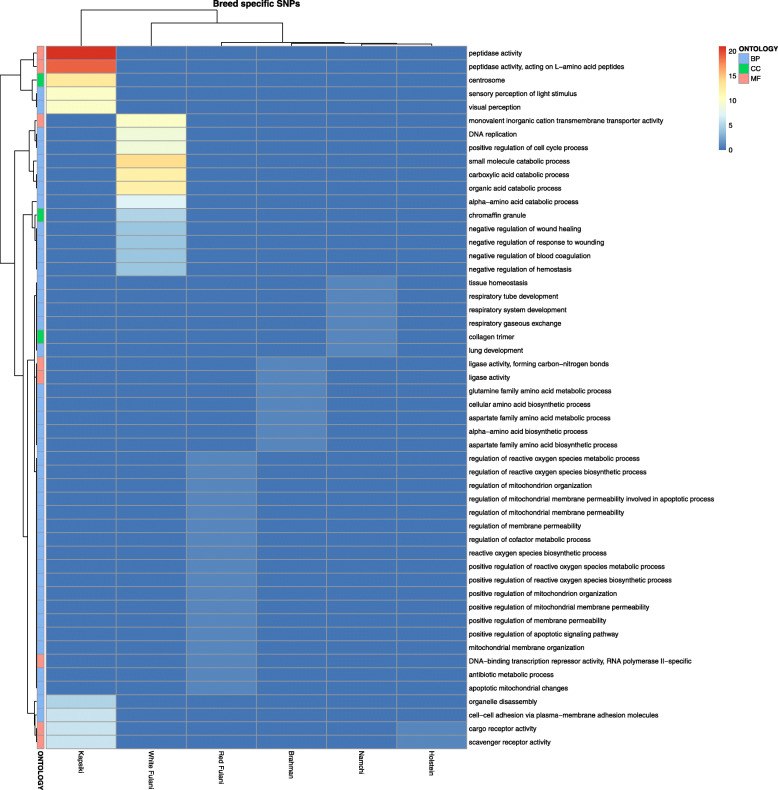
Variant Gene Ontology (GO) of novel, missense and breed-specific variants. **A)** Heat map of gene ontology terms of different cattle breed-specific SNPs of high and moderate impact. **B**) Heat map of gene ontology terms of different cattle breed-specific InDels of high and moderate impact. The GO terms belonging to biological processes (BP), cellular components (CC) and molecular functions (MF) are shown in red, green and blue, respectively. The color of each cell indicates the number of variant carrying genes

These genes were then used to perform a Gene ontology (GO) enrichment analysis for each breed separately. Fifty-two significantly enriched GO terms were identified (Fig. 5) with most significantly enriched terms derived from Kapsiki and White Fulani. Those breeds showed the highest number of genes with bs-SNPs and bs-InDels of high and moderate impact. Interestingly, we found enrichment of GO terms related to adaption to high-altitude environment and heat tolerance in Kapsiki, namely the GO terms “peptidase activity” and “scavenger receptor activity”. In Namchi, although such GO terms were not significantly enriched, genes such as *ADAMTSL1*, an *ADAMTS* like gene, *OR9G1*, an olfactory receptor (OR) and the surfactant protein *SFTPD* were identified carrying either missense variants or frameshift variants (Additional file 3 Table S3). These genes are often reported in the context of heat stress via their interaction with heat shock proteins, but have been also often reported in the context of wound healing [29, 30]. This would imply some genes that contribute to important resistance traits are passed onto hybrid offspring in the Namchi, and might therefore be interesting candidates for further research on increasing trypanotolerance in WAS. In contrast, several enriched GO terms were found in the trypano-susceptible African zebu cattle White and Red Fulani which were absent in the taurine breeds Kapsiki and Namchi. Those GO terms were linked to a negative regulation of wounding or wound healing (Fig. 5, for White Fulani) and a positive regulation of the mitochondria and reactive oxygen species (ROS) metabolic processes (Fig. 5, for Red Fulani). Mitochondria are an important source of ROS within most mammalian cells. They are also generated at wound sites, and act as long-range signals in wound healing. Hence, controlling genes associated with these GO terms might play a vital role in the adaptation to infectious diseases in Zebu cattle breeds. In Gudali, another trypanosusceptible African zebu cattle breed, no enriched GO terms were found for SNPs and InDels (Fig. 5). However, several missense variants of high impact were found on an Interferon-inducible GTPase gene (Additional file 3 Table S7). These GTPases provide host resistance to a variety of viral, bacterial and protozoan pathogens through the sequestration of microbial proteins, manipulation of vesicle trafficking, regulation of antimicrobial autophagy [29, 30], which are all congruent for a significant role in the adaptation to infectious diseases.

Taken together, the functional annotation and Gene Ontology analysis identified breed-specific high and moderate impact variants as genetic traits which could help explaining cattle-breed specific phenotypes, such as heat tolerance in Kapsiki and trypanotolerance in Namchi.

### Adaptation to tropical climate and high altitude

Adaptation to local environment is multifactorial involving several genes [1–3]. To cope with heat, poor food and high altitude, African cattle have developed behavioral, cellular and physiological responses to mechanical stress, oxygen, food deprivation and homeostasis [31]. During the evolution of Zebu cattle, they have acquired genes for heat-tolerance at the physiological and cellular levels [32]. The superior ability for regulation of body temperature during heat stress is the result of lower metabolic rates as well as increased capacity of heat tolerance. Heat stress also leads to lightening of the coat, because light colored hair coats have a sleek and shiny reflection [32]. However, the lower metabolic rates under heat stress condition are related to reduction in feed intake, milk yield, thyroid hormone secretion, and growth. This finding may explain the lower performance of meat growth in African Zebu cattle as compared to taurine breeds of European descent. Among many other genes involved in heat stress, four heat shock factor (HSF) genes (HSF1, HSF2, HSF3, and HSF4) have been isolated in vertebrates. HSF1, which is located on chromosome 14, is a master regulator of Heat Shock Protein (HSP70) expression during heat shock [33]. Its interaction with the heat shock proteins HSPA1A/HSP70 or DNAJB1 result in the inhibition of heat shock- and HSF1-induced transcriptional activity during the attenuation and recovery phase from heat shock [34, 35]. European taurine Holstein, WAS, WAZ and Indian Zebu Brahman cattle possess distinct patterns of homozygosity and heterozygosity for the SNP alleles of HSF1. The heterozygosity alleles in these genes were over-represented in WAS and WAZ as compared to Brahman and Holstein. The increased heterozygosity among the African cattle breeds (WAS and WAZ) indicates the combined effects of genetic isolation and long selection history. In addition, when looking at high and moderate impact bs-SNPs only, in the trypanosusceptible breeds Kapsiki (WAS) and White Fulani (WAZ), but not in the trypanotolerant Namchi and N’Dama, mutations in heat shock proteins were found. The observation that some trypanosusceptible Zebu breeds such as White Fulani carry many mutations in heat shock protein encoding genes [see Additional file 3 Table S6] while other trypanosusceptible Zebu breeds such as Red Fulani and Gudali do not carry any mutations in heat shock proteins, could be further investigated in future genomics research of African cattle breeds towards improving heat stress resistance of those cattle breeds.

### Adaptation to pathogens

Stress response, olfactory receptors and immune responses play a critical role in adaptation to the tropical environment and diseases [17, 25]. Mammalian olfactory receptors (Ors) are encoded by the largest mammalian multigene family with more than 1000 genes organized in clusters on 26 cattle chromosomes [36]. They are essential for avoiding danger, food search, reproduction, and behaviour [36]. Ors have been linked to heat stress but were also reported to accelerate wound healing [29, 37–39], where chemokines play a critical role by enabling the phagocytic leukocytes of the immune system to be the first line of defense against infectious agents, such as protozoa and helminth parasites [30]. The tolerance of Namchi cattle against trypanosomiasis (trypanotolerance) caused by the protozoan parasites *Trypanosoma congolense*, *T. vivax* and *T. brucei* is actively driven by the innate immune response. IL-12, INF-γ and TNF-α are primarily produced by cells of the innate immune system and trigger phagocytic cell activation and inflammation, thus contributing to the control of parasite growth [40]. Sialic acid binding immunoglobulin-like lectin (SIGLEC) and the major histocompatibility complex (MHC) gene family, also known in cattle as bovine leukocyte antigens (BoLA) genes, are key players involved in the regulation of chemokines and cells of innate and adaptive immune responses. SIGLECs are expressed on various white blood cells of the immune system and are involved in the regulation of innate and adaptive immunity [41]. In contrast, studies have also shown that many coated sialylated viruses, bacteria and parasites are capable to mimic self-recognition and thus dampen or evade an immune response [41].

Genetic polymorphisms in the mentioned genes have been often linked to wounding processes and pathogen resistance. For instance, polymorphisms in BOLA-DRB3 and other BoLA genes were linked to resistance against viral, bacterial and other parasite infections [42–45]. In this study, 6 high and moderate impact variants such as frameshift or missense variants were detected in BoLA genes in Kapsiki, and 9 were found in White Fulani. Furthermore, high and moderate impact variants were found in Namchi, located in Ors such as the previously mentioned *OR9G1* gene and in the *SIGLEC-1* gene in Kapsiki (Additional file 3, Table S3 and S5). In addition, a different level of polymorphisms in the genomic region of the BoLA genes on chromosome 23 of the cattle reference genome was observed for all breeds analyzed (Additional file 4, Fig. S11). The trypanosusceptible cattle Kapsiki carried the highest number of homozygous and heterozygous alleles in this region while the other studied trypanotolerant cattle breeds such as Namchi and N’Dama showed a lower level of polymorphism, especially in the BOLA-DYB and BOLA-DOB regions (Additional file 4, Fig. S11). However, the results obtained here for Namchi could also be due to previously mentioned possible hybrid status of the selected animal with a recent introgression of Zebu genes into its genome.

The findings provide the first comprehensive set of genome-wide high quality sequencing and variant data of the most important Cameroonian cattle breeds. Although this study was conducted on single samples per breed only, which does not allow us to correctly separate within from across breed variation, we think that the obtained high quality genomic data shall constitute a foundation for breed amelioration whilst exploiting the heritable traits and support conservation efforts for the endangered local cattle breeds.

## Conclusions

The whole genome of five indigenous Cameroonian cattle Namchi, Kapsiki, Gudali, White Fulani and Red Fulani was sequenced and analyzed for the first time, and variant calling results were compared to the reference genomes of European *Bos taurus* Holstein, African *Bos taurus* N’Dama and one Asian Zebu *Bos indicus* Brahman. The findings obtained in this study indicated that both Namchi and Kapsiki cattle possess genotypes and phenotypes associated with disease susceptibility or resistance and heat tolerance, which are complex mechanisms involving several gene pathways located on different chromosomes. This is in line with previous findings, and therefore the high impact variants found in this study could provide potential markers for future genome-wide association studies (GWAS). All the candidate genes could hence constitute a valuable resource for development and genetic amelioration of tropical cattle breeds, particularly in Africa. Furthermore, the full high depth sequence data widens our knowledge on the value of native breeds as genetic resources for future cattle breeding, and the power of selection signature analyses.

## Methods

### Sampling, library construction and sequencing

The data used for this paper was obtained from the project “Pathogen detection in African cattle Breeds” Abanda et al. [18] and Paguem et al. [19].

One representative individual of each of the five different cattle breeds was selected (Table 3).

**Table 3:** Information of the selected animals of Cameroonian cattle breeds for whole genome re-sequencing

Breed	Age [years]	Sex	Sampling sites		GPS Coodinates		Altitude	LW [kg]	Subspecies
			Region	Village	N	E			
Namchi	6	male	Faro	Herko	8°30'05.1''	13°08'28.7''	520m	252	*Bos taurus brachyceros*
(Doayo)									
Kapsiki	5	female	Mayo-Tsanaga	Rhumsiki/Kila	10°27'45.5''	13°38'22.9''	956m	252	*Bos taurus brachyceros*
W. Fulani	5	female	Mayo-Rey	Bini	07°37'29.6''	14°32'10.1''	780m	240	*Bos indicus indicus*
R. Fulani	5	female	Mayo-Rey	Bini	07°37'29.6''	14°32'10.1''	780m	313	*Bos indicus indicus*
Gudali	7	female	Vina	Galim	07°12'2.39''	13°34'49.70''	1050m	400	*Bos indicus indicus*

*W. Fulani* White Fulani, *R. Fulani* Red Fulani, *LW* Live weight

Blood samples of 5 ml volume per animal were collected in ethylene diamine tetra acetic acid (EDTA)-coated vacutainers during the routine examination. The blood was centrifuged at 3000 rpm for 15 min. Then, genomic DNA was extracted from the buffy coat (cellular layer including leucocytes, erythrocytes and blood-dwelling parasites like Anaplasmataceae bacteria, piroplasmids, microfilariae of *Setaria*, trypanosomes and *Borrelia* spp. (see Additional file 5 Table S12 for trypanosome, *Onchocerca* filarial and gastrointestinal parasites detected on those animals) using the Wizard Genomic DNA Purification Kit (Promega, Germany) according to the manufacturer’s instructions. DNA isolation and concentration was verified by fluorescent methods using Picogreen (Life Technologies). Libraries were generated from 2 μg of genomic DNA per specimen using the Illumina TruSeq DNA PCR-Free Library Prep Kit (Illumina, San Diego, CA, USA) following the manufacturer’s protocol. 2 × 150 bp paired-end libraries sequencing was conducted on the Illumina HiSeq4000 platform with the manufacturer’s proprietary TruSeq SBS Kit V3-HS.

### Short read mapping, variant calling and annotation

The quality of the generated raw Illumina reads was determined using Fast QC software (http://www.bioinformatics.bbsrc.ac.uk/projects/fastqc/). Adaptor read sequences were removed using SeqPurge from ngs-bits4 (https://github.com/imgag/ngs-bits, version 0.1–4-gaed0c94). For comparison with other cattle breeds, whole genome raw sequencing data from NCBI Sequence Read Archive SRA was extracted for the breeds Holstein (SRR934414), N’Dama (SRR3693376) and Brahman (SRR6649996). Paired-end reads from the five samples along with these three controls from the SRA archive were mapped against the reference *Bos taurus* Hereford breed genome UMD3.1 (NCBI refSeq accession GCA_000003055.5) and ARS-UCD1.2 (NCBI RefSeq accession GCF_002263795.1) respectively, using BWA-MEM version 0.7.10-r789 [41]. Reads that mapped to a single location in the genome (uniquely mapped reads) were selected, and those with multiple region mapping were excluded using the MarkDuplicates tool of Picard5 v.1.137 (http://broadinstitute.github.io/picard). After sequence alignment, the resulting SAM files format were converted to BAM files using Samtools v.1.3 [46]. Then BAM files were sorted and local realignment of reads was performed to correct misalignment due to the presence of small InDels using Genome Analysis Tool Kit 3.1 (GTAK). SNPs and InDels calling were performed using Freebayes v.0.9.21–19-gc003c1e [47]. SNPs and InDels were annotated using snpEFF [28] and Bcftools [46]. To have many of these processes parallelized and automated, a workflow written in the workflow language Snakemake from QbiC was used which is freely available at Github (https://github.com/qbicsoftware/exomseq).

The variants that were identified in only one cattle breed and have no corresponding entries in the dbSNP database (for mapping to UMD3.1) or 1000 Bull Genomes Project (for mapping to ARS-UCD1.2) were classified as breed-specific novel variants. The average ratios of homozygous versus heterozygous SNPs were calculated for each breed. This ratio is expected to be 1:2 in a freely mating population; therefore, any departure from this condition such as the presence of admixture in the population will be manifested by an increase in the homozygous/heterozygous ratio [48].

### Unmapped read analysis

Reads that were not mapped to the reference genome UMD3.1 and ARS-UCD1.2, respectively, were extracted from alignment BAM files and sorted by name using Samtools. The sorted BAM files were given as input to AbySS (version 2.1.5) and assembled using the parameter “k = 25” indicating k-mer size = 25 in standard de Bruijn graph mode. Resulting contigs.fa files were subdivided into contigs with a length > 500 bp. Then the remaining contigs were searched against Blastn database using Nucleotide-Nucleotide BLAST (version 2.8.1+) with the parameters “-num_alignments 1”and “-num_descriptions 1” to show alignments and descriptions for the top 1 matching database match only. The BLAST output was then parsed using the R language (version 3.4.0) to determine for each sample the species of the BLAST hit, the percent identity, length of match and query, and BLAST e-value. Mean values of these statistics were calculated for each species in each sample.

### Gene enrichment and functional analysis

For downstream analysis of single nucleotide polymorphisms (SNPs) we used the genome ARS-UCD1.2 but not UMD3.1 and compared it with reference genomes of European *Bos taurus* Holstein, Asian *Bos indicus* Brahman and African trypanotolerant N’Dama breeds. Breed-specific non-synonymous (bs-ns) SNPs, InDels with moderate and high impact in the genome and new variants not found in any publicly available database were extracted from WAS and WAZ using the data repositories Ensembl release 76, dbSNP138, Entrez Gene, NCBI and Uniprot. The variant carrying genes were functionally characterized based on different gene ontology (GO) terms using clusterProfiler (v3.12) R package(v3.5.2) [49].

### Phylogeny of bovine-related species

To understand the genetic relationships between indigenous cattle breeds and other subfamilies of Bovidae, a principal component analysis (PCA) was performed with EIGENSTRAT. For the phylogenetic tree reconstruction, the variant files were converted to FASTA format with Vcf-kit8 (https://vcf-kit.readthedocs.io/en/latest/). Multiple sequence alignment (MSA) was generated using Muscle with default options [50]. Prottest3 [51] was used to find the best substitution model for the MSA, and RAxML was used to generate the Maximum Likelihood (ML) tree with Blossum62 as best substitution model along with Gamma distribution for rate heterogeneity, estimation for proportion of invariable sites and 100 non-parametric bootstrap replicates using Brahman as outgroup [52]. Visualization of the tree was generated using ape (v5.3) R package [53].

## Supplementary information

**Additional file 1: Table S1.** Pairwise alignment of contigs assembled from unmapped reads to the non-redundant nucleotide database.

**Additional file 2: Figure S2.** Distribution of SNPs, InDels and breed-specific SNPs per chromosome and breed. Bar plot illustrates the number of SNPs found in at least two breeds (green), breed-specific SNPs (blue) and InDels (orange) across all the breeds for each chromosome.

**Additional file 3: Table S3.** High impact Namchi breed specific SNP. **Table S4.** High impact Kapsiki breed specific SNP. **Table S5.** High impact Red Fulani breed specific SNP. **Table S6.** High impact White Fulani breed specific SNP. **Table S7.** High impact Gudali breed specific SNP. **Table S8.** High impact Brahman breed specific SNP. **Table S9.** High impact Holstein breed specific SNP. **Table S10.** High impact N’Dama breed specific SNP.

**Additional file 4: Figure S11.** Distribution of SNPs per cattle breed of chromosome 23 between location 25,350,340 and 25,593,072 containing the BoLA gene. The X axis represents genomic location and y-axis represents ratio of non-reference base. Value 1 indicates that all reads carry the non-reference base at a given location whereas a value of 0.5 and 0 indicates half and none of the reads carry the non-reference base, respectively.

**Additional file 5: Table S12.** Trypanosome, *Onchocerca* filarial and gastro-intestinal parasites detected from five animals of each cattle breed detected by microscopy and molecular diagnostics using ribosomal nuclear makers.

## Data Availability

All data generated or analyzed during this study are included in this published article, and its additional information files are available from the corresponding author on reasonable request. The five newly sequenced African cattle genomes in this study are publicly available from GenBank with the Bio project SRA accession: PRJNA596606).

## Abbreviations

GO: Gene ontology
PCA: Principal component analysis
WAS: West African Shorthorn
WAZ: West African Zebu
bs-ns: Breed-specific non-synonymous
HSPA: Heat Shock 70 Kda protein
HSF: Heat shock factor
Ors: Olfactory receptors
BoLA: Bovine leucocyte antigen
SNPs: Single nucleotide polymorphism variants
InDels: Insertions and Deletions variants
Gb: Giga base pairs
