# Supplementary figures and images for: Whole genome characterization of autochthonous Bos taurus brachyceros and introduced Bos indicus indicus cattle breeds in Cameroon regarding their adaptive phenotypic traits and pathogen resistance
Source: BMC Genet. 2020 Jun 22;21:64. doi: 10.1186/s12863-020-00869-9 (PMC7309992; doi:10.1186/s12863-020-00869-9)

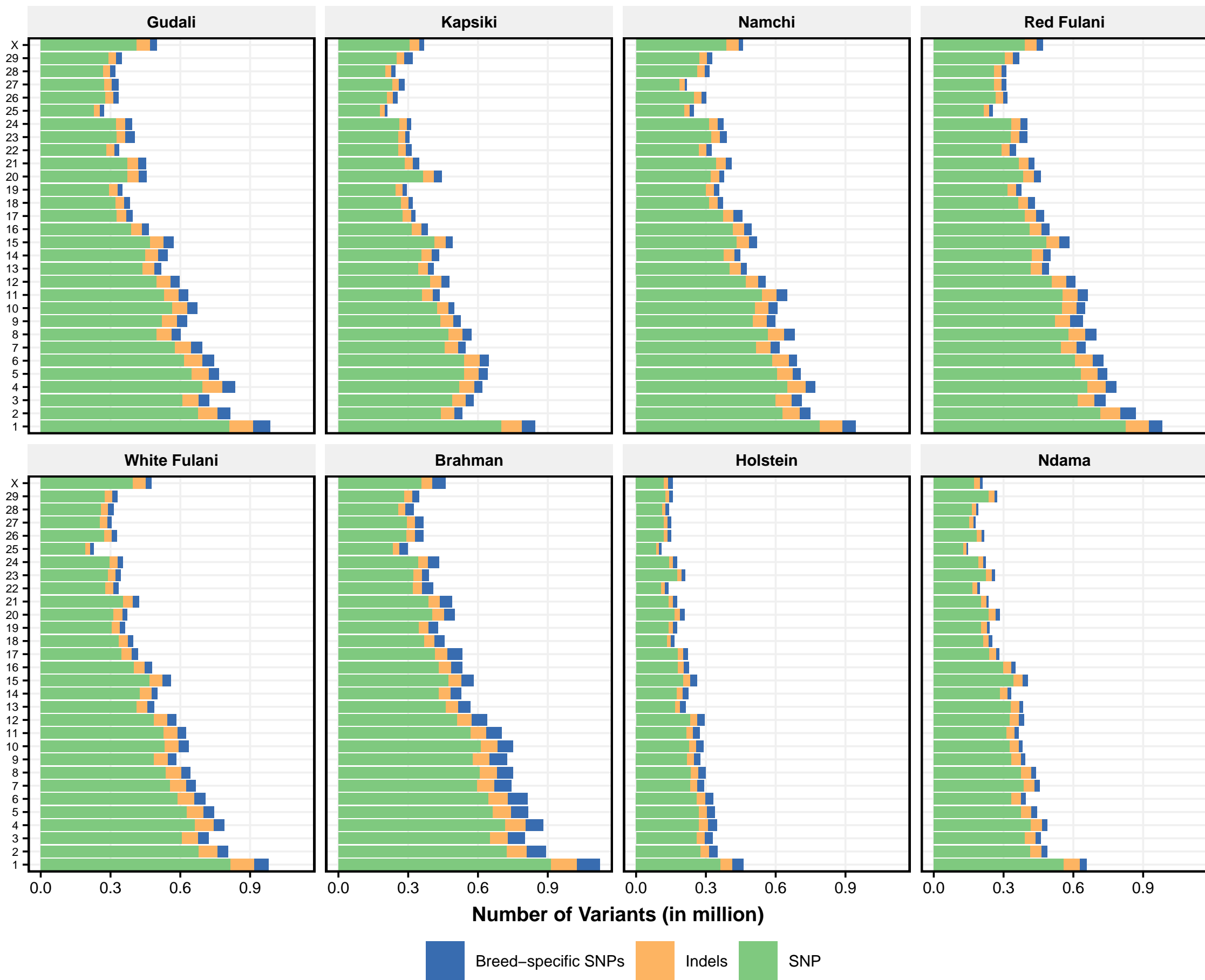

Supplement: Supplementary file 2 — Additional file 2: Figure S2. Distribution of SNPs, InDels and breed-specific SNPs per chromosome and breed. Bar plot illustrates the number of SNPs found in at least two breeds (green), breed-specific SNPs (blue) and InDels (orange) across all the breeds for each chromosome. [file 12863_2020_869_MOESM2_ESM.pdf]

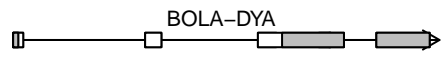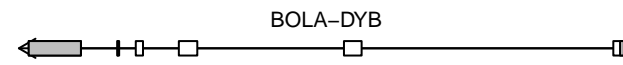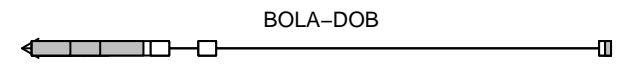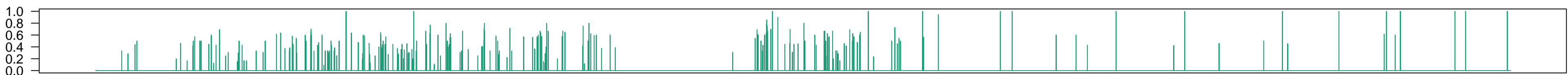

**Namchi**

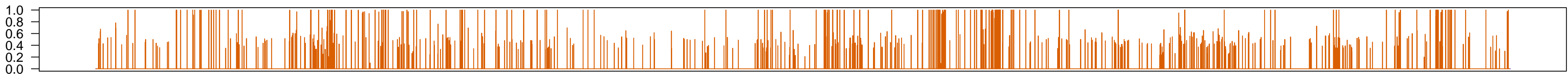

**Kapsiki**

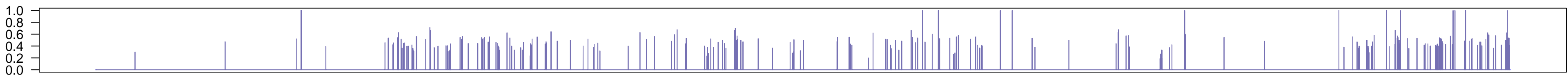

**White Fulani**

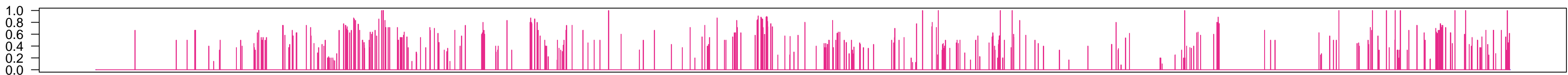

**Red Fulani**

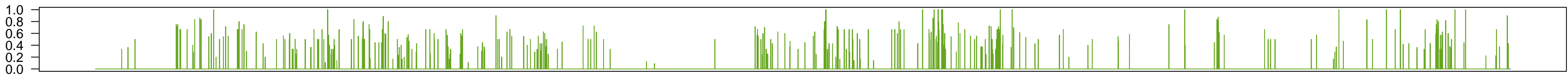

**Gudali**

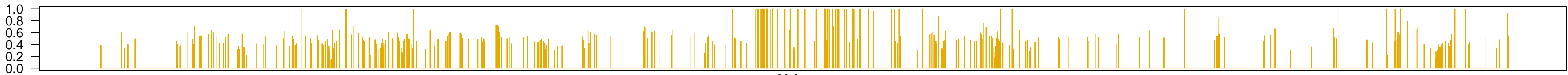

**Ndama**

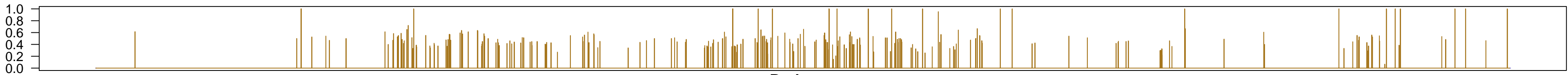

**Brahman**

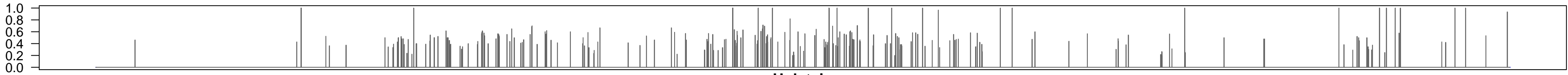

**Holstein**

Supplement: Supplementary file 4 — Additional file 4: Figure S11. Distribution of SNPs per cattle breed of chromosome 23 between location 25,350,340 and 25,593,072 containing the BoLA gene. The X axis represents genomic location and y-axis represents ratio of non-reference base. Value 1 indicates that all reads carry the non-reference base at a given location whereas a value of 0.5 and 0 indicates half and none of the reads carry the non-reference base, respectively. [file 12863_2020_869_MOESM4_ESM.pdf]
